# Benefits of Early Thoracic Surgery in the Elderly During the COVID-19 Pandemic: Lessons Learned From Performing a Video-Assisted Thoracotomy

**DOI:** 10.7759/cureus.31461

**Published:** 2022-11-13

**Authors:** Aldin Malkoc, Tammy Phan, Danny T Nguyen, Harpreet Gill, Alexander T Phan, Jaclyn R Cerceo, Albert Nguyen, Olga Lebedevskiy, Bruce Toporoff

**Affiliations:** 1 Department of General Surgery, Arrowhead Regional Medical Center, Colton, USA; 2 Department of General Surgery, Kaiser Permanente Fontana, Fontana, USA; 3 School of Medicine, California University of Science and Medicine, Colton, USA; 4 Department of Internal Medicine, Arrowhead Regional Medical Center, Colton, USA; 5 Department of Cardiothoracic Surgery, Loma Linda University Medical Center, Loma Linda, USA; 6 Department of Cardiothoracic Surgery, Arrowhead Regional Medical Center, Colton, USA

**Keywords:** psycho-social, video-assisted thoracoscopic surgery (vats), elderly trauma, mini thoracotomy, covid 19, sars-cov-2

## Abstract

Elderly patients are often considered poor surgical candidates for intra-thoracic operations due to the number of comorbidities, increased risks associated with general anesthesia, decreased cardiopulmonary reserve, and overall increased frailty. In addition, coronavirus disease 2019 (COVID-19) is a critical psychosocial factor that, through secondary effects, can prevent patients from receiving optimal care. Patients are reduced to having limited contact with family, often a vital support system, which can contribute to feelings of hopelessness, loneliness, and depression. We report the case of a 95-year-old female who presented to the emergency department with increasing supplemental oxygen requirements two weeks after a ground-level fall. She was found to have multiple rib fractures and a left-sided hemothorax. Initial management included aggressive respiratory therapy, multiple pigtail chest tubes, and thrombolytics; however, these measures failed to drain the intrathoracic hematoma. Her care was complicated by the psychosocial and isolation factors of COVID-19 which led to the patient exhibiting symptoms of hopelessness, grief, lack of appetite, and loneliness. As conservative management did not improve her clinical care the patient required a video-assisted thoracoscopic surgery (VATS) to manage the retained hemothorax and facilitate re-expansion of her atelectatic lung. Once the patient was removed from COVID-19 precautions, she was taken to surgery and postoperatively the patient reported minimal pain, participated more in physical therapy, and increased her oral intake. In this unique case, a 95-year-old patient with a hemothorax that was successfully treated with a VATS had her clinical care complicated by the psychosocial implications of COVID-19.

## Introduction

Elderly patients are often considered to be poor surgical candidates for intra-thoracic operations due to multiple chronic medical conditions, risks of general anesthesia, decreased cardiopulmonary reserve, and increased frailty [1]. Prolonged hospitalization is a stressful event for elderly patients, leading to functional decline, worsening disability, and mortality [2]. While the medical complications from coronavirus disease 2019 (COVID-19) have been extensively described, the psychosocial complications in the elderly are less emphasized. Romero et al. suggested that active severe acute respiratory syndrome coronavirus 2 (SARS-CoV-2) infection can lead to more profound muscular weakness in elderly patients on bed rest, which can rapidly reduce functional status in this population [3]. Acute hospitalization in the elderly population and immobilization has been associated with significant potential harm [4]. In a study of 60 functionally independent individuals 75 years or older admitted to the hospital from their home for acute illness, 75% were no longer independent on discharge, including 15% who were discharged to nursing homes [5]. Therefore, elderly patients during the COVID-19 pandemic represent a unique population that has the potential to benefit from early surgical intervention to initiate earlier physical therapy and discharge. In particular, elderly patients requiring thoracic surgery and a COVID-19 diagnosis require special considerations.

Video-assisted thoracoscopic surgery (VATS) is a minimally invasive, safe, and reliable option to perform various diagnostic and curative procedures [6,7]. VATS is considered integral to the early management of thoracic trauma, including retained hemothoraces and persistent pneumothoraces [7,8]. It allows for visualization and evacuation of a hemothorax, while preventing further injury to the chest wall, preventing decortication of empyemas, and providing the ability to address persistent air leaks [7]. The intraoperative and postoperative morbidity has been cited to be less than 10%, and mortality has been shown to be under 1-2% [6]. Furthermore, earlier thoracic surgical intervention can reduce length of hospital stay when compared to conservative treatments [9-11]. In particular, careful and rapid management of traumatic retained hemothorax from blunt chest trauma provides improved outcomes [7]. In a study of 78 patients greater than 65 years of age with blunt chest trauma, an earlier VATS procedure showed an average four-day reduction in ICU stay and an eight-day reduction in hospital stay [11]. Additionally, elderly patients aged 80 to 91 with a delayed presentation to thoracic surgery have been noted to have an increased mortality of 6.6% [12]. In elderly patients, the concerns of prolonged hospitalization and functional decline warrant consideration of early operative treatment. This case report describes a 95-year-old female with rib fractures complicated by hemothorax and a superimposed SARS-CoV-2 infection with prolonged hospitalization and functional decline. She underwent a successful video-assisted thoracotomy (VAT) after conservative measures failed to resolve a retained hemothorax.

## Case presentation

**Figure 1:**
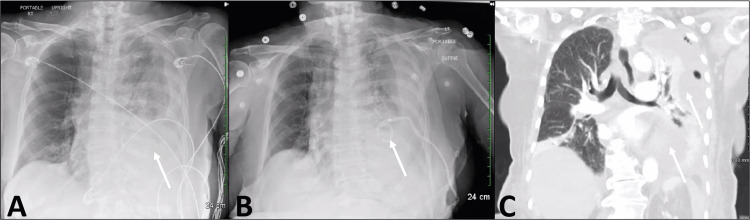
A). Anterior-posterior chest x-ray on admission showing a left-sided chest pleural effusion indicated by the white arrow. B). Hospital day 13 anterior-posterior chest x-ray showing minimal improvement after administration of tissue plasminogen activator and dornase alfa, white arrow indicating the pigtail catheter through which the tissue plasminogen activator and dornase alfa was given. C). Coronal segment of a computed tomography of the chest showing complete atelectasis and a left-sided hemothorax as indicated by the two white arrows.

A 95-year-old COVID-19-vaccinated female with a reported ground-level fall two weeks prior to admission was brought from an independent nursing facility to the hospital for increasing dyspnea. The initial physical exam was significant for mild distress, left posterior chest wall tenderness, decreased breath sounds on the left hemithorax, and normal right-sided breath sounds. Incentive spirometry performed yielded a result of approximately 100mL. Point-of-care ultrasound was performed and showed absent lung sliding and hypoechoic lung segments. Chest radiography demonstrated a large left-sided pleural effusion (Figure 1A). A complete blood cell count (CBC) showed a leukocytosis of 13,000 cells/μL (Normal = 4,300-11,000 cells/μL), hemoglobin of 10.9 g/dl (Normal = 13-17 g/dl). Basic metabolic panel was within normal limits except a potassium level of 5.4 mEq/L (Normal = 3.5-5.5 mEq/L). Coagulation tests were within normal limits. A urine drug screen (Roche Diagnostics, Indianapolis, IN) was negative. Computed Tomography (CT) without contrast of the chest revealed a large left-sided pleural effusion and heterogeneous hyperdensity in the lower hemithorax, as well as posterolateral ninth and 10th rib fractures. The patient was admitted to the surgical intensive care unit (SICU) for close hemodynamic monitoring.

On hospital day 1, the patient underwent a left-sided tube thoracostomy, which drained approximately 700mL of dark, clotted blood. On hospital day 6, the left chest tube drained minimal amounts (<100mL) of serosanguinous fluid and the hemothorax had resolved. The chest tube was removed, and a subsequent chest x-ray showed no hemothorax; however, a left pleural effusion with increased size was noted. At this time, the patient mentioned difficulty maintaining an appetite and admitted to feelings of despair and depression due to her persistent weakness and difficulty ambulating. On hospital day 11, the patient was tachycardic with a leukocytosis of 22,000 cells/L (Normal = 4,300-11,000 cells/μL). She was started on empiric antibiotics of cefepime 1g every 12 hours and a five-day course of 500mg azithromycin. Repeat thoracic CT imaging demonstrated increased consolidation of the left lung space and required ultrasound-guided drainage of the suspected left chest hemothorax with a 12 French pigtail catheter (Figure 1B). Prior to her procedure, the patient was tested for SARS-CoV-2 and noted to be positive.

**Figure 2:**
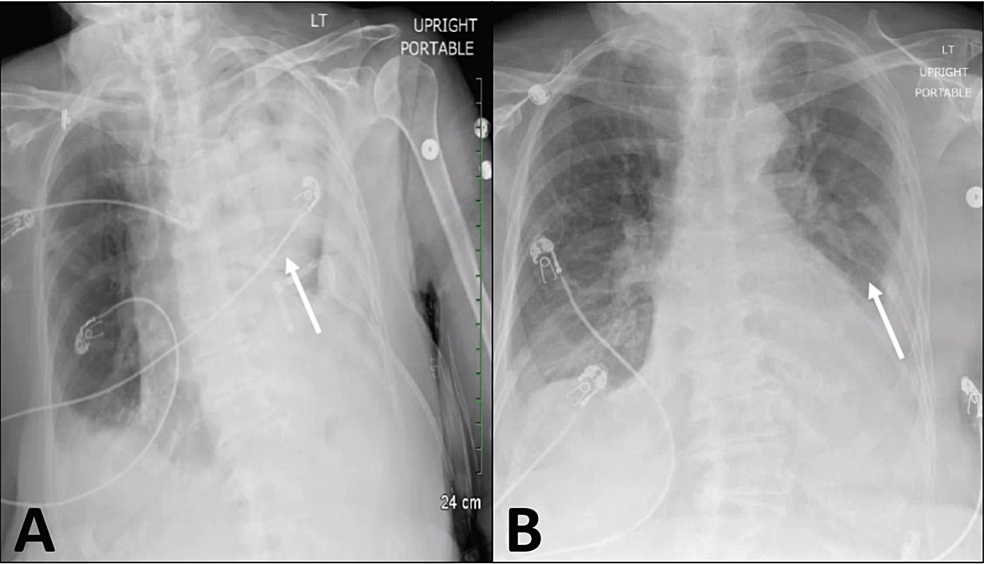
A). Anterior-posterior chest x-ray prior to scheduled video-assisted thoracostomy showing bilateral congestion and large left effusion, indicated by the white arrow. B). Anterior-posterior chest x-ray taken after video-assisted thoracostomy showing near resolution of the left-sided effusion and full left lung expansion, white arrow indicating the normal lung parenchyma.

On hospital day 13, the patient was noted to be developing worsening leukocytosis with an increase to 23,500 (Normal = 4,300-11,000 cells/μL). There was minimal improvement in the left-sided consolidations, so tissue plasminogen activator (TPA) and dornase alfa were administered through the left-sided pigtail catheter in attempts to augment drainage. After administration, the patient’s chest tube output increased and chest x-ray on hospital day 17 showed decreased left-sided pleural effusion with improvement in her leukocytosis to 15.5× 109/L. On hospital day 20, a CT of the chest with IV contrast was obtained, as the pigtail catheter drained only 20mL in the previous 24-hour period, with no further improvement in chest X-ray. On hospital day 23, our interventional radiology (IR) specialist attempted to place a new pigtail catheter in the left upper hemithorax, but the procedure was unsuccessful with an iatrogenic injury from the procedure to the lung resulting in a nearly completely atelectatic left lung, shown in Figure 1C. On hospital day 24, the patient’s oxygen saturation on pulse oximetry dropped to 80%, requiring high flow nasal canal. Cardiothoracic surgery was consulted at this time for a VATS, with possible left thoracotomy. Figure 2A shows the chest x-ray prior to the planned VATS with a large left effusion.

Left-sided video assisted mini thoracotomy was performed through a 6 cm incision in the left sixth interspace without rib resection. A significant amount of serosanguinous fluid and old clots were found within the thoracic cavity. Additionally, there were significant dense adhesions trapping the left lung, requiring decortication of the left lower lobe. The thoracoscope allowed visualization of the entire pleural cavity, and superior clots, as well as clots in the major fissure, were removed. Approximately 700mL of combined serosanguinous fluid with old and fresh clots were evacuated, and the left lung was fully re-expanded. A chest tube was then placed in the left anterior chest.

Postoperatively, the patient was admitted to the SICU for close monitoring for a duration of three days until she was transferred to a telemetry unit. A chest x-ray after the video-assisted thoracotomy showed resolution of the left-sided effusion and full left lung expansion (Figure 2B). The chest tube output had less than 100mL of serosanguinous fluid over the subsequent 24 hours. The patient’s incentive spirometry improved to >750mL, and in the immediate postoperative period, she required only 1-2 liters of supplemental oxygen to keep oxygen saturation levels above 92%. The patient reported minimal pain, participated more in physical therapy, and increased her oral intake. The patient was taken off COVID-19 isolation parameters following our hospital’s isolation guidelines and her family visited her regularly. She suffered no complications in the immediate postoperative period and was transferred to the medical-surgical unit on postoperative day 3.

## Discussion

Conservative measures are often the first-line treatment in elderly patients who present with thoracic trauma that may be amenable to surgical treatment. This is usually due to hesitation about the possible intraoperative or postoperative complications that may arise due to comorbid conditions. In this case, definitive surgical treatment was delayed, specifically because of her advanced age and SARS-CoV-2 infection, which caused significant psychological strain on the patient. COVID-19 is a critical social factor that, through secondary effects, can prevent patients from receiving optimal care. Patients have limited contact with family, often a vital support system, which can contribute to feelings of hopelessness, loneliness, and depression. SARS-CoV-2-positive patients are often isolated from nursing and physical rehabilitation staff and often impair early mobilization and recovery [13]. Loss of taste and smell can decrease oral intake and negatively impact nutritional status, which can lead to impaired wound healing [14]. A strong body of evidence exists about the negative impacts of social isolation. There is a known link between social isolation and disease-related mortality. In addition, multiple studies have demonstrated that social isolation is an independent predictor of all-cause mortality [15].

During the patient’s COVID-19 isolation precautions, she continued to express feelings of depression and displayed lack of willingness to participate in physical activity, often complaining about the inability to see her family and her overall condition. During her conservative management, the patient required oxygen therapy and expressed excessive tiredness. A recent study by Barnes et al. of nearly 8000 patients suggested social isolation and loneliness were associated with increased mortality in the elderly population [16]. After the patient’s VATS, she was removed from COVID-19 precautions and had improved functional breathing status; subsequently, she began to participate more in physical therapy and became more invested in her overall plan of care. Additionally, as her family was able to visit more frequently, she noted decreased feelings of depression and improvement in her appetite. COVID-19 has significant and serious complex medical concerns, but the psychosocial factors in the elderly population warrant serious consideration. The patient’s definitive treatment with VATS provided earlier physical rehabilitation and, once removed from COVID-19 isolation precautions, there was a noted improvement in emotional factors as well.

Early surgical intervention should be considered in all patients, especially the elderly, and particularly in those who are under COVID-19 isolation precautions. VATS can be used to safely perform a wide variety of procedures in selected elderly patients, as there is a low complication rate and association with shortened hospital length of stay [17]. The risks of delaying minimally invasive thoracic surgery are marginal when compared to the risks associated with social isolation and prolonged hospitalization. In this patient who was positive for SARS-CoV-2, the delay in definitive surgery led to multiple pigtail catheter drain placements and a prolonged hospital stay, which ultimately led to physical and social isolation for several weeks prior to definitive surgical treatment with VATS.

## Conclusions

In this unique case, a 95-year-old patient underwent a video-assisted thoracotomy after unsuccessful conservative management of a hemothorax that was complicated by COVID-19. COVID-19 was a significant social factor that negatively affected her overall condition and her care. Surgical intervention, when deemed appropriate, should be offered in a timely manner, particularly in elderly patients struggling with the social isolation that accompanies a diagnosis of COVID-19.
